# Design of a structure with low incident and viewing angle dependence inspired by *Morpho* butterflies

**DOI:** 10.1038/srep03427

**Published:** 2013-12-05

**Authors:** Wanlin Wang, Wang Zhang, Jiajun Gu, Qinglei Liu, Tao Deng, Di Zhang, Hai-Qing Lin

**Affiliations:** 1 State Key Laboratory of Metal Matrix Composites Shanghai Jiao Tong University 800 Dongchuan Road, Shanghai, P. R. China; 2 Beijing Computational Science Research Center, Beijing, P. R. China

## Abstract

*Morpho* butterflies *are* well known for their brilliant iridescent colors, which arise from periodic arrays of scales. These brilliant colors have a low angle dependence, in contrast to similar phenomena that are commonly caused by the periodic structures. We designed a structure with a low incident and viewing angle dependence inspired by *Morpho* butterflies. This structure was studied using the finite-difference time-domain method. The lamellae distribution of tree-like structure was found to be the determining factor for producing a low incident angle dependence. Two advanced models were designed to produce a low viewing angle dependence. Model I was constructed using two layers of scales. The particle swarm optimization algorithm was used to construct Model II. The angle dependence of Model II exhibited a large viewing angle range under various incident angles.

The structural color of photonic crystals can be tuned by changing the periodicity of the structure or the refraction index of the material, without the use of color filters or additional optical elements. Functional materials with structural color have wide range applications, such as in full color paper-like displays, packaging, and advertising. However, the change in hue associated with changing the incident or viewing angle for these photonic crystals can pose major challenges in the development of color displays with wide viewing angles^1^. Colors vary with the viewing angle because the resonance condition changes as the incident light direction varies with respect to the crystal orientation. This variation in color with angle is well-understood. However, structural color that is not strongly angle-dependence is less well understood and has been less exploited. In this study, we designed a structure with a wide viewing angle range at large incident angles based on the structure of *Morpho* butterfly wings.

In this study, we investigated one type of structural color in nature to understand and explore low angle dependence. Many species in nature exhibit structural color, including peacock feathers, buprestid elytron, and *Morpho* butterfly wings. The blue color of the wings of the *Morpho* butterfly is one of the examples of iridescent coloration with high reflectivity and low angle dependence. The *Morpho*-blue has been attributed to grating and multilayer structures^2^,^3^,^4^,^5^,^6^,^7^. The high reflectivity of the blue color can be explained by interference among the layers. However grating and multilayer structures produce a high angle dependence, whereas the structural color in *Morpho* butterflies exhibits a much smaller angle dependence. This low angle dependence is not observed in other manifestations of structural colors in nature such as soap bubbles, beetles^8^,^9^ and birds' feathers^10^,^11^ which show rainbow-like, shimmering iridescence. Thus, the mysterious color of the *Morpho* butterfly must arise from a more complex structure. In this paper, complex models were constructed to describe the structural color of the *Morpho* butterfly. Some but not all, of the features of the mysterious color are explained.

Several studies have been published on the broad angle dependence of the structural color of *Morpho* butterfly wings based on measurements^12^,^13^,^14^,^15^,^16^,^17^,^18^,^19^ and theoretical analyses^19^,^20^,^21^,^22^,^23^. The angle dependence has been described in detail^18^,^19^: when viewing a wing from a direction perpendicular to the wing veins, the wing color can suddenly appear as violet or dark blue at a sufficiently large viewing angle; however, when the wing is viewed from a direction parallel to the wing veins, the blue color abruptly vanishes and the wing appears black. It has also been reported that the random distribution of heights of ridges eventually results in a cancellation of the interference among neighboring ridges, resulting in diffusive reflection, as though each ridge was scattering light independently. A randomized structure has been invoked in some reports^20^,^22^,^24^ to explain why sharp peaks are not observed. The wide angular reflective distribution was found to be determined by variations in the lamellae under normal incidence^20^,^22^. We achieved a larger viewing angular range using particle swarm optimization (PSO) algorithm to optimize as well as randomize the lamellae distribution^23^.

Most studies on angle dependence have been conducted for normal incident angles^20^,^22^,^23^,^24^. However, various incident angles have been investigated in some studies^13^,^25^. The visible change in color from blue to deep blue to deep violet upon varying incident angles from 0 to 45° has been demonstrated using a numerical model^13^. Angular measurements and simulations have been performed^25^ on *Morpho rhetenor,* showing that the optical properties of this butterfly can be well characterized by retroreflection in two inverse directions under oblique incidence. Although the *Morpho* butterfly has already been studied and analyzed fairly intensively, it is necessary to simulate a more realistic model under various incident angles.

Most species of the *Morpho* butterfly (e.g., M. *didius*) have two types of scales, ground scales and cover scales^26^. The cover scales determine the wing appearance and the reflectivity of the iridescent wing^18^,^19^. In a previous report, a model was constructed by combining a ground scale and a cover scale^27^, but angle dependence was not explored. In this paper, Model I was constructed by combining a ground scale and a cover scale to produce low angle dependence.

For a regular lamellae distribution, a tree-like structure produces the same angle dependence as a grating structure^23^. In practice, the lamellae distribution in natural *Morpho* butterflies is not regular. Random lamellae distributions exhibit a lower angle dependence than regular lamellae. Low angle dependence can also be produced^23^ using a PSO algorithm. In this paper, Model II was constructed by varying ΔL(lamellae distribution) for a simple tree-like structure.

The PSO algorithm, was inspired by the social behavior of animal species, such as birds, bees, and others, and was introduced by Eberhart and Kennedy^28^, as a search tool. The PSO algorithm has recently served as a robust, stochastic evolutionary strategy for solving electromagnetic problems^29^,^30^ and has been used in the design of nonlinear and non-continual optimization approaches with continuous variables. PSO is also easy-to-implement in the design of optical diffraction gratings^31^. In this study, the PSO algorithm was used to optimize the angle dependence, which is a nonlinear optimization problem^23^.

We produced an incident angle dependence for a tree-like structure that was lower than that of a multilayer structure and found the influential parameter responsible for this difference. Two mechanisms of butterflies (cover scales and lamellae distribution) were investigated to obtain a low viewing angle dependence. Our goal was not to create an exact computational model for the *Morpho* butterfly but rather to study the angle dependence of structural color in *Morpho* butterflies and to design a structure with a wide viewing angle range at large incident angles.

Models for *Morpho* butterfly wings have become increasingly complex, evolving from simple multilayers^5^ and multilevel gratings^32^ to distributions of dielectric multilayers^12^ and tree-like structures^22^,^33^,^34^,^35^,^36^. We studied the microstructure of *Morpho* scales using a finite-difference time-domain (FDTD) method. Geometrical data were obtained from previous studies. The *Morpho* microstructure was modeled in this study as a tree-like structure, which is schematically shown in Figure 1. We assumed that the refractive index of chitin was 1.56 + 0.06i, in accordance with results in the literature^12^,^22^,^34^, and that the remaining regions were filled with air with a refractive index of 1.0. We denote the incident angle and viewing angles by α and β, respectively, as shown in Figure 1(a). The tree-like structure originated from a multilayer structure, as shown in Figure 1(b). The following multilayer parameters were used defined as: lamella height = 60 nm, lamellae interval = 140 nm and number of layers = 5. Three important parameters of the tree-like structure are shown in Figure 1(b): ΔR (width of ridge) = 60 nm, ΔT (lamellae taper) = 10 nm and ΔL (lamellae distribution) = 0 nm, 100 nm, 0 nm, 100 nm, …. The same ΔL was used for each ridge for simplicity. Most of the aforementioned geometrical data were obtained from electron microscopy studies^12^,^37^. These parameters have been confirmed in previous studies^20^,^33^,^34^,^35^,^36^. Only three parameters ΔR, ΔT and ΔL are discussed here, being the identifying parameters of the tree-like structure compared with the multilayer structure, as shown in Figure 1(b).

## Results

### Derivation of low incident angle dependence of structural color in Morpho butterfly wings

The blue color of *Morpho* butterfly wings can be explained very well at normal incidence angles in terms of a multilayer structure, as shown in Figures 2(a)(c). We can see that a reflection peak at an incidence angle of 0° located in a blue region approximately 450 nm. The high incident angle dependence of the multilayers can also be observed in Figures 2(a)(c). The following conclusions can be drawn. (1) The positions of the reflection peaks exhibit a high incident angle dependence under s and p polarization, and the peaks move to shorter wavelengths when the incident angle increases under both polarizations. (2) The peak intensities of the reflections exhibit a high incident angle dependence under p polarization. The peak intensities decrease when the incident angle increases. Thus, the multilayer structure is invisible at large incident angles.

A simple explanation for the incident angle dependence of a tree-like structure can be provided in terms of two multilayer phenomena. The blue shift in Figures 2(a)(c) can be understood using Eq. (1), which is based on the theory of multilayer interference^18^. 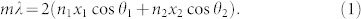
In Eq. (1), *m* is an integer, *n* is the refractive index, *x* is the layer thickness, subscript 1 denotes air and subscript 2 denotes chitin. The angles of refraction in the air and chitin layers are denoted by *θ*_1_ and *θ*_2_ respectively. When α increases from 0° to 60°, *θ*_1_ (which is equal to α) also increases, and thus *θ*_2_ increases. As a result, λ is shifted to shorter wavelengths as shown in Figures 2(a)(c).

Figure 2(i) shows the reflection and refraction of natural light, which illustrate why the peak intensities under p polarization show a high incident angle dependence. Increasing the incident angle causes more p-polarized light to be refracted and therefore results in low reflection. When the incident angle equals Brewster's angle, reflection of the p polarization is not observed at all. In this case, Brewster's angle equals α = 57°. Thus, the reflections of the p polarization are almost equal to 0 for an incident angle above 57°, as shown in Figure 2(c). This difference is clearly shown in Figures 2(e) and (f), where an electric field intensity contour plot is displayed for an incident angle of 60° and a wavelength of 440 nm.

Let us compare the strong angle dependence of the multilayers to the lower angle-dependent reflection of a simple tree-like structure, as shown in Figures 2(b)(d). (1) The peaks of the reflections have a lower incident angle dependence, and the blue shifts of the multilayers shown in Figures 2(a)(c) under s and p polarization almost disappear. (2) The peak intensities of the reflections under the p polarization have a low incident angle dependence. Although the peak intensities for large incident angles are still lower than those for small incident angles, the difference between the intensities is smaller for the tree-like structure than for the multilayer structure. Figures 2(g) and (h) illustrate this result in terms of an electric field intensity contour plot for an incident angle of 60° and a wavelength of 440 nm for a simple tree-like structure.

**Figure 1:**
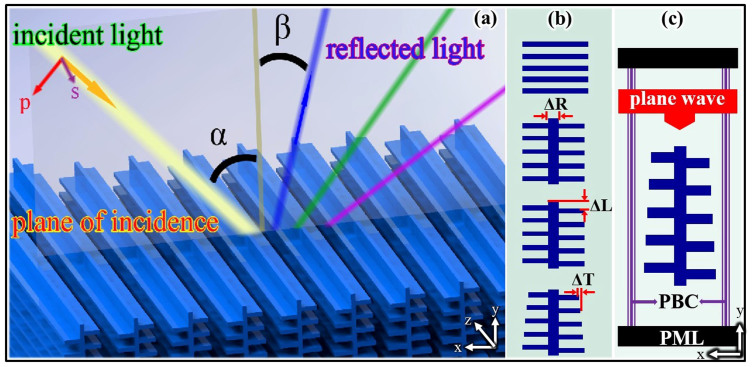
Computational model. (a) tree-like structure, incident light, reflected light, incident angle α, and viewing angle β; and (b) evolving the model from a multilayer to a tree-like structure by incorporating three parameters: ΔR (ridge width) = 60 nm, ΔT (lamella taper) = 10 nm, and ΔL (lamella distribution) = 0 nm, 100 nm, 0 nm, 100 nm, ….; (c) the boundary condition in the vertical direction is absorbing (perfectly matched layer, PML), and the boundary condition in the horizontal direction is periodic (periodic boundary condition, PBC).

The aforementioned results show that a simple tree-like structure can reduce the incident angle dependence. A simple-tree like structure was developed from a multilayer structure by incorporating the following parameters: ΔR (ridge width), ΔL (lamella distribution), and ΔT (lamella taper), which are shown in Figure 1(b). These three parameters were investigated to find the determining parameter affecting the incident angle dependence, and the results are shown in Figure 3.

**Figure 2:**
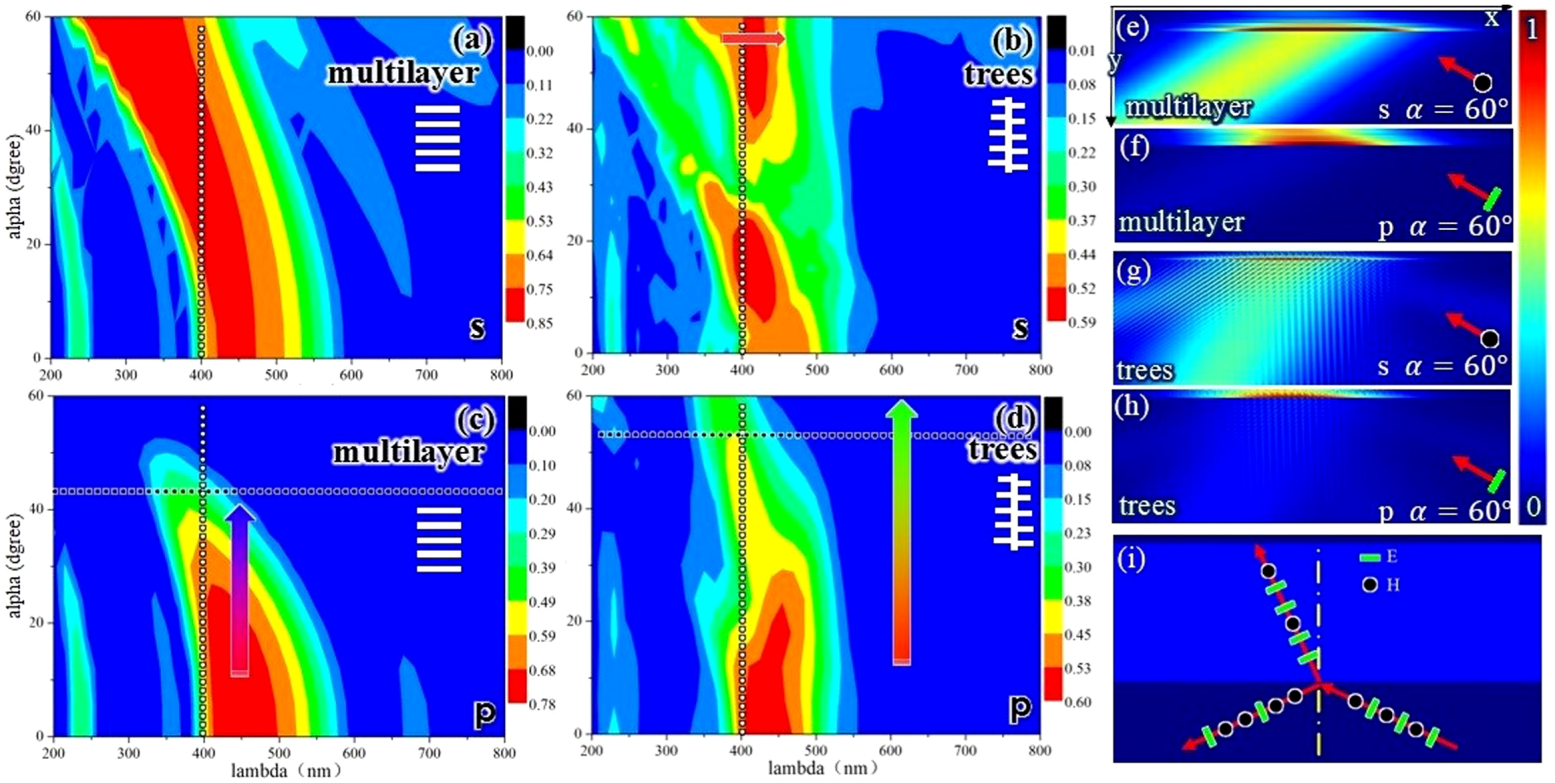
Incident angle dependence of multilayer and tree structures. (a–d) contour plot showing reflections versus wavelength and incident angle (α) for multilayer and tree-like structures under s and p polarization (the color-coded intensities correspond to a linear scale); (e–h) electric field intensity contour plot for multilayer and tree-like structures under s and p polarization for incident angle α = 60° and wavelength λ = 440 nm (the color-coded intensities correspond to a linear scale); and (i) reflection and refraction of natural light; the arrows in (b),(c), and (d) show the shift of the reflective peak or the trend in the reflective intensity; x and y are defined in Figure 1.

**Figure 3:**
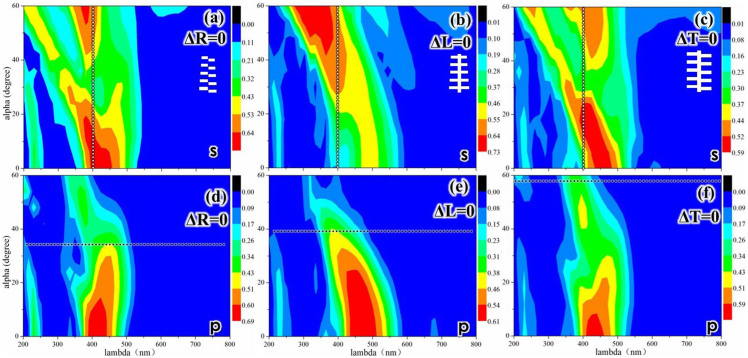
Contour plot showing reflections versus wavelength and incident angle (α) of a tree-like structure. (a), (b), and (c) ΔR = 0 nm, ΔL = 0 nm, and ΔT = 0 nm under s polarization; (d), (e), and (f) ΔR = 0 nm, ΔL = 0 nm, and ΔT = 0 nm under p polarization ( the color-coded intensities correspond to a linear scale).

Figure 3 shows a contour plot of the reflections versus the wavelength and the incident angle for ΔR, ΔT, and ΔL equal to 0 nm. The results are summarized here. (1) Figures 3(c) and (f) show that when ΔT equals 0 nm, the reflection is still enhanced for a large incident angle under p polarization, and the blue shift is not observed under either polarization. Thus, ΔT does not cause the low angle dependence. (2) Figures 3(b) and (e) show that when ΔL equals 0 nm, the reflection is suppressed for a large incident angle under p polarization, and the blue shift can be clearly observed under both polarizations. These phenomena were also observed for a multilayer structure, as shown in Figures 2(a) and (c). Thus, ΔL is a determining factor in the lower angle dependence of a tree-like structure. (3) Figures 3(a) and (d) show that when ΔR equals 0 nm, the reflection is still enhanced for large incident angles under p polarization, and the blue shift is still observed under both polarizations; however, the enhancement and the magnitude of the shift are smaller than for the simple tree-like structure shown in Figures 2(b) and (d). Thus, ΔR can impact the incident angle dependence but has a smaller effect than ΔL.

Thus, the parameter ΔL (lamella distribution) can be considered to be the determining factor in reducing the incident angle dependence. In previous studies, ΔL was found to be the determining factor for the viewing angle dependence^18^,^20^,^23^, thus ΔL greatly affects both the incident angle and the viewing angle dependence.

After investigating the low incident angle dependence of a simple tree-like structure, we will discuss the viewing angle dependence of this structure in the next section.

### Using a simple tree-like structure to produce reflections with sharp peaks

**Figure 4:**
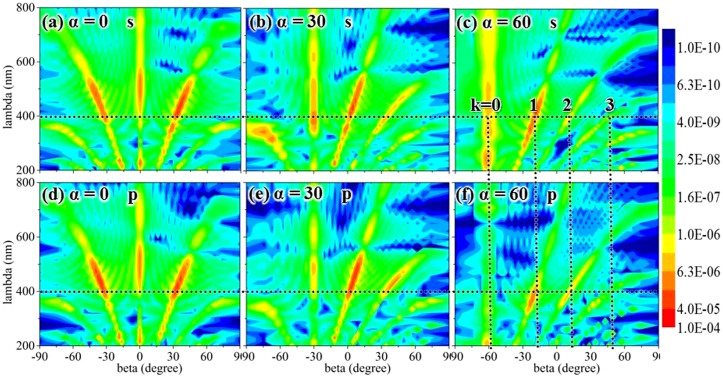
Contour plot showing reflections versus wavelength and viewing angle (β) of a tree-like structure. (a), (b), and (c) incident angles α = 0°, 30°, and 60°, respectively, under s polarization; (d), (e), and (f) incident angles α = 0°, 30°, and 60°, respectively, under p polarization; “k” is the diffraction order (the color-coded intensities correspond to a logarithmic scale).

We computed the reflective angular dependence of a simple tree-like structure for monochromatic illumination at incidence angles of 0°, 30°, and 60°, which correspond to the far-field scattered intensities. The far-field was calculated using a distance of 7500 nm = 10*T (where T = 750 nm is the period of a single tree-like structure). Figure 4 shows reflections with very sharp peaks, which agree well with grating theory. 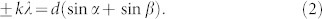
We calculated peaks at α = 0°, 30°, and 60° from the grating formula in Eq. (2) for a wavelength λ = 400 nm and a grating constant d = 750 nm. These theoretical grating values agree very well with the simulation results. Only the results for α = 60° are discussed here. When the diffraction order “k” was 0, 1, 2, and 3, the peaks were located at −60°, −19°, 11°, and 47°, respectively, showing good agreement with Figures 4(c) and (f). It has been reported^23^ that under normal incidence, the viewing angle dependence of a simple tree-like structure is equivalent to the viewing angle dependence of a grating structure^23^. In terms of the viewing angle dependence, a tree-like structure can be considered to be a grating structure for any incident angle. The reflections under s and p polarization were found to have exactly the same peak locations as shown in Figure 4, but the intensities were different. The differences between these intensities agree with the results shown in Figure 2. Thus, the polarization does not impact the peak position of the viewing angle of a simple tree-like structure, but it does impact the intensity of the viewing angle.

The spectrum of viewing angles shown in Figure 4 exhibits very sharp peaks corresponding to grating theory. These sharp peaks are enhanced by the multilayer structure of the lamellae. Within optical theory, it would be reasonable to assume that a microstructure evolved from the tree-like structure produced the sharp peaks. However, the presence of these sharp peaks contradicts the low viewing angle dependence of the natural *Morpho* blue color. In this study, we did not reproduce the exact structure of the Morpho butterfly wing and all of its optical properties, such as a smooth spectrum of reflection; however, we did reproduce some interesting features of the structural color of the Morpho butterfly: the large viewing angle range and low incident angle dependence.

In the following section, we describe the development of two complex models developed from a simple tree-like structure for the realization of our objective. Model I was constructed by adding cover scales to ground scales. Model II was constructed by varying the parameter ΔL (lamella distribution) using the PSO algorithm. These two models were generated to enlarge the viewing angular range under various incident angles.

### Derivation of low viewing angle dependence of the structural color in Morpho butterfly wings

**Figure 5:**
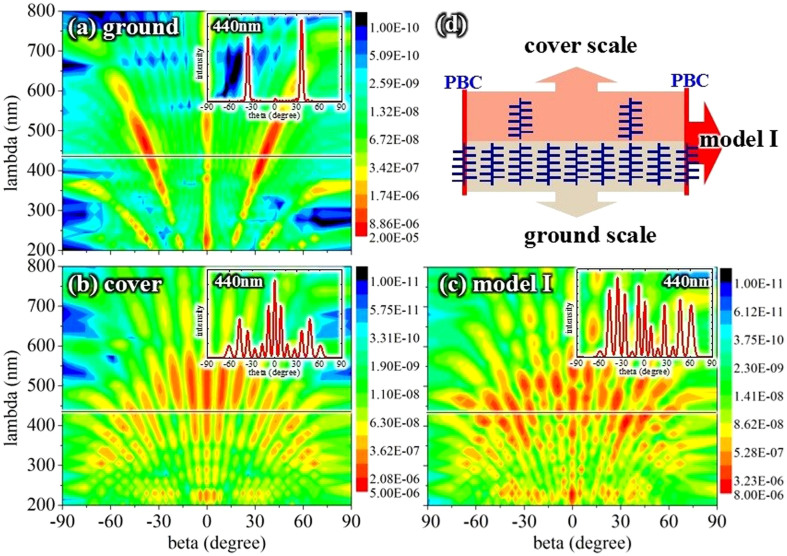
Construction of Model I. (a–c) contour plot showing reflections versus viewing angle and wavelength for ground scales, cover scales, and Model I, respectively (the color-coded intensities correspond to a logarithmic scale); and (d) schematic of Model I, which is a combination of cover and ground scales.

Microstructural observations of the arrangement of the *Morpho* wing scales, including the horizontal surface and cross-section, were used to construct Model I, which combines both the ground and cover scales, as shown in Figure 5(d). The ground scale was modeled as the simple tree-like structure shown in Figure 1(b), and the cover scale was modeled as the same tree-like structure but with a period of 4*T, following a study in the literature^17^. This model is not a precise mathematical model for the cover and ground scales of the *Morpho* butterfly but serves to determine the parameter controlling the angle dependence. The far-field scattered intensities were calculated using a distance of 2*4*T = 6000 nm, and the results were compared with the simulation results shown in Figure 4, which were calculated for a distance of 10*T.

#### Normal incidence

The reflections of the ground scales shown in Figure 5(a) agree with the results shown in Figure 4(a). For T = 750 nm, ΔL = 100 nm, and wavelength = 440 nm, the reflection of diffraction order k = 1 is enhanced and the reflection of k = 0 is suppressed according to grating theory. The cover scales have a smaller viewing angle dependence than the ground scales, although the intensity of the cover scales is much lower, as shown in Figures 5(a) and (b). Figure 5(c) shows the viewing angle dependence of Model I, which is a combination of cover and ground scales. The results show that the viewing angle dependence of Model I is smaller than that of the ground scale and even smaller than that of the cover scales. This result confirms the reported conclusion^18^,^19^ that one of the functions of the cover scales is to reduce the viewing angle dependence. The insets in Figures 5(a), (b), and (c) show line plots of the reflection versus the viewing angle for a wavelength of 440 nm, which clearly show the low viewing angle dependence of Model I under normal incidence. Thus, Model I can produce a larger viewing angular range than a simple tree-like structure under normal incidence.

#### Various incident angles

**Figure 6:**
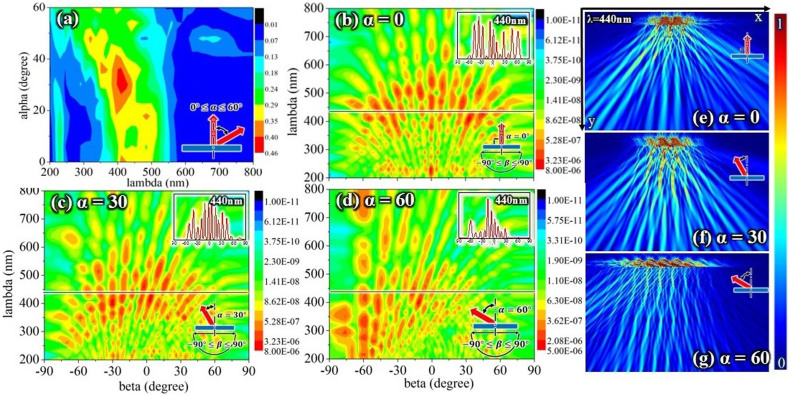
Low angle dependence of Model I. (a) contour plot showing reflections versus wavelength and incident angle for Model I (the color-coded intensities correspond to a linear scale); (b–d) contour plot showing reflections versus viewing angle and wavelength for Model I at incident angles of 0°, 30°, and 60° (the color-coded intensities correspond to a logarithmic scale); and (e–g) electric field intensity contour plot for Model I for incident angles of 0°, 30°, and 60° and a wavelength of 440 nm (the color-coded intensities correspond to a linear scale); x and y are defined in Figure 1.

Figure 6(a) shows that Model I produces a greater dependence on the incident angle than a multilayer structure. Figures 6(b)–(d) show the viewing angle dependences at incident angles of 0°, 30°, and 60° using the calculated far-field scattered intensities for a distance of 2*4*T = 6000 nm. The results show that the viewing angle dependences at incident angles of 0°, 30°, and 60° are much lower than those obtained for multilayer and simple tree-like structures. The insets in Figures 6(b)–(d) show that for a wavelength of 440 nm, the dependence at an incident angle of 0° is lower than that at an incident angle of 60°. Figures 6 (e)–(g) show the low viewing angle dependence more clearly at incident angles of 0°, 30°, and 60° using an electric field intensity contour plot. Thus, cover scales can produce a low viewing angle dependence under various incident angles but cannot produce a low incident angle dependence. The results also show sharp peaks in the reflections. Thus, the cover scales can enlarge the viewing angle range but cannot smooth out the sharp peaks.

Cover scales represent one type of feature that impacts the angle dependence of the structural color of the *Morpho* butterfly wing. Another feature that impacts the angle dependence is the lamella distribution (ΔL). In the discussion above, the variation in the lamella distribution (ΔL) was held fixed for simplicity. In practice, every lamella is different; but the distribution of every lamella deviation is different and irregular in real *Morpho* butterflies. The PSO algorithm has been used to investigate this problem for normal incidence^23^. Thus, the PSO algorithm was used to construct Model II to study the incident and viewing angle dependence at various incident angles.

#### Normal incidence

**Figure 7:**
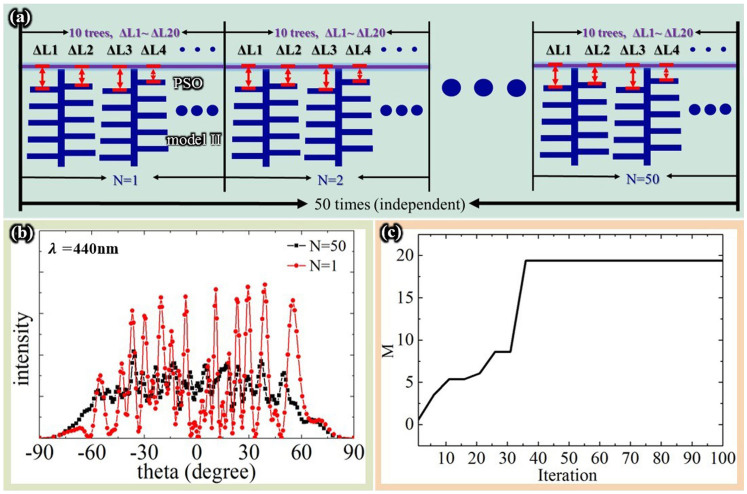
Constructing Model II. (a) schematic of Model II using the PSO algorithm; 10 trees are simulated by adjusting ΔL1 ~ ΔL20 for each case; the algorithm is run 50 times, and the results are averaged; (b) reflections versus viewing angle for N = 1 and N = 50; and (c) fitness value versus number of iterations for N = 1.

Figure 7(a) shows a schematic of Model II that was constructed using the PSO algorithm. Different lamella distributions were defined using a parameter Δy. Ten trees were simulated for each distribution: thus, the variable Δy took values from Δy1 to Δy20. The values for Δy varied from −60 nm to 60 nm. After completing simulations for a total of N = 50 cases, the results were averaged, as in a previous study^20^. We defined a fitness function M as the ratio of the variance of the peak intensities to the variance of the peaks of the viewing angles. This function was used to characterize the viewing angle dependence.

Figure 7(b) shows the low viewing angle dependence derived from the far-field scattered intensities, which were calculated for a distance of 10*T = 7500 nm. The reflection of N = 1 in Figure 7(b) shows that varying ΔL in the PSO algorithm produced a wide viewing angular range. However, the result for N = 1 also shows that the peaks of the viewing angle remain very sharp. To smooth out the sharp peaks, we performed 50 optimization runs and averaged the results. Figure 7(b) shows the average result for the 50 cases: although some peaks remain, the reflection is smoother than the result for N = 1. Thus, we conclude that different parameters are required to produce a large viewing angular range and a smooth spectrum. A large viewing angular range can be obtained by varying ΔL in the PSO algorithm.

Figure 7(c) reports the values of M versus the iteration number, showing that the M curve converged after 37 iterations. The optimized ΔL values for N = 1 were ΔL1, ΔL2, …, ΔL20 = [−60 nm, 60 nm, 25 nm, 60 nm, 16 nm, −60 nm, 60 nm, 19 nm, −35 nm, −60 nm, 60 nm, 60 nm, −60 nm, 60 nm, 60 nm, 25 nm, 60 nm, 57 nm, −60 nm, −60 nm]. Model II was constructed using these ΔL values.

#### Various incidence

**Figure 8:**
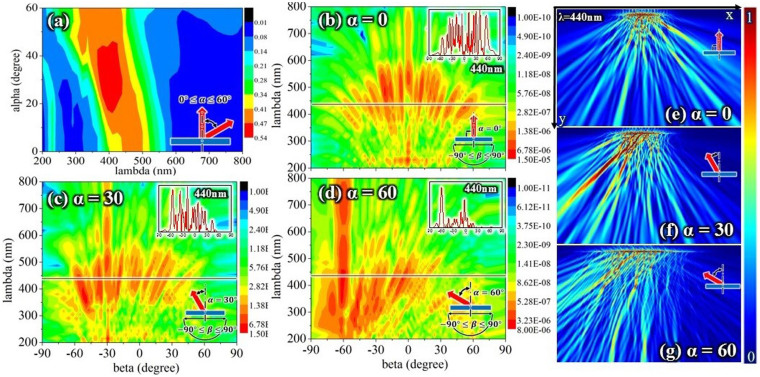
Low angle dependence of Model II. (a) contour plot showing reflections versus wavelength and incident angle for Model II; (b–d) contour plot showing reflections versus viewing angle and wavelength for Model II at incident angles of 0°, 30°, and 60° (the color-coded intensities correspond to a logarithmic scale); (e–g) electric field intensity contour plot for Model II at incident angles of 0°, 30°, and 60° and a wavelength of 440 nm (the color-coded intensities correspond to a linear scale); x and y are defined in Figure 1.

Figure 8(a) shows the incident angle dependence of Model II. The incident angle dependence of Model II is clearly weaker than that obtained for Model I and the simple tree-like structure. The intensities of the peaks under various incident angles are all greater than 40%. Figures 8(b)–(d) show the viewing angle dependence of the far-field scattered intensities, which were calculated for a distance of 10*T = 7500 nm. The results show a low viewing angle dependence at various incident angles. The insets in Figures 8(b)–(d) clearly show a low viewing angle dependence for a wavelength = 440 nm, and the dependence at an incident angle of 0° is lower than that at an incident angle of 60°. Figures 8 (e)–(g) show the low viewing angle dependence at incident angles of 0°, 30°, and 60° more clearly, using an electric field intensity contour plot.

### Future work

In this study, we designed a structure exhibiting a wide viewing angle range for a large incident angle range, inspired by the structure of *Morpho* butterfly wings. The structure must be sufficiently ordered, as in multilayer and grating structures, to produce the desired color and reflectivity. At the same time, the structure must also be sufficiently disordered, both in terms of its shape and location, to remove the directionality and sharp reflectance peaks associated with multilayered interference.

The models presented here can be used to guide artificial fabrication processes. The incident angle dependence of Model II is weaker than that of Model I. An appropriate model should be chosen to fit the application at hand; however, we believe that both models can be artificially fabricated.

## Discussion

The low angle dependence at various incident angles of the tree-like structure of *Morpho* butterfly wings was studied using FDTD analysis. The low incident angle dependence observed in a simple tree-like structure can be explained by comparing its reflection with that of a multilayer structure. The parameter ΔL (lamella distribution) was shown to play an important role in reducing the incident angle dependence. (1) The blue shift in the reflections of the multilayer was affected by variations in the parameter ΔL. (2) The reflection was enhanced at a large incident angle under p polarization upon variations in the parameter ΔL. The peak positions and peak intensities exhibited by a simple tree-like structure were smaller than those for a multilayer. The parameter ΔL was shown to cause this difference. The controlling factors of *Morpho* butterflies were changed to reduce the viewing angle dependence. One factor was the cover scale structure (which was explored in Model I). This result confirms the reported conclusion^18^,^19^ that one of the functions of the cover scales is to reduce the viewing angle dependence. The other is to adjust parameter ΔL(lamellae distribution) by using PSO algorithm (model II). Under various incident angles, the angle dependence of Model II exhibited a larger viewing angular range but still exhibited sharp peaks. Model II was constructed N times, and the results were averaged. The averaged curve was much smoother than the result for a single construction. The features of Models I and II are most likely both important for describing the structural color in butterfly wings; however, we expect Model II to be more significant than Model I. These two models both demonstrate low viewing angle dependence at various incidence angles.

## Methods

The reflectivity was calculated using an FDTD method, which is one of the most commonly used techniques for solving the scattering problem of periodic dielectric structures. A planar wave light source was used to study the angle dependence: the boundary condition in the vertical direction was a perfectly matched layer (PML), and a periodic boundary condition (PBC) was used in the horizontal direction, as shown in Figure 1(b). A Gaussian wave was used to simulate the electric field (as in Figures 2(e)–(h)), and the boundary conditions were PML in both the vertical and horizontal directions. To ensure that the simulations were accurate at various incident angles, we tuned the wavelength from 200 nm to 800 nm and the incident angles from 0° to 60°. The mesh size was chosen to obtain a good tradeoff between the computer memory required and the simulation time, while ensuring convergence of the results. A convergence test was carefully performed.

## Author Contributions

W.L.W. and W.Z. contributed equally to this work and performed the FDTD simulations; J.J.G., Q.L.L. and T.D. helped with data analysis and theoretical calculations. D.Z. and H.Q.L. performed the optimizations. W.L.W., W.Z. and T.D. contributed to the writing of the manuscript.
